# ‘*At Least I Get to Visit Him, That's All That Matters*’: Maintaining Contact With a Family Member in Prison, Developing a Child‐Centred Framework for Change

**DOI:** 10.1111/hex.70625

**Published:** 2026-03-25

**Authors:** Naomi Griffin, Lisa Crowe, Nancy Loucks, Shona Minson, Tracy Shildrick, Tina Young, Steph Scott

**Affiliations:** ^1^ Population Health Sciences Institute Newcastle University Newcastle Upon Tyne UK; ^2^ Families Outside Edinburgh Scotland UK; ^3^ Strathclyde, Centre for Law, Crime & Justice University of Strathclyde Glasgow Scotland UK; ^4^ Centre for Criminology University of Oxford Oxford UK; ^5^ School of Geography, Politics and Sociology Newcastle University Newcastle Upon Tyne UK; ^6^ Nepacs Durham UK

**Keywords:** children, co‐design, families, health & wellbeing, imprisonment, inequalities, prison visits, qualitative research, young people

## Abstract

**Introduction:**

Familial imprisonment is one of ten recognised adverse childhood experiences (ACE), with established long‐term impacts on health, care and wellbeing. Where safe and appropriate, the right of a child to protect and maintain family life (and therefore visit and/or remain in contact with a family member in prison) is protected in the United Nations Convention on the Rights of the Child (UNCRC). Despite this, families face many barriers when visiting prison, and children and young people's experiences of doing so, in their own words, are less widely reported.

**Methods:**

Drawing on serial longitudinal interviews and the curation of creative methods with 19 children and young people (age 7–16) who have a family member in prison across Northern England and Scotland, the aim of this study was to explore the impact of familial imprisonment on children and young people's health and wellbeing, and to utilise our findings to develop a rights‐based framework for prison social visits.

**Results:**

Reflexive thematic analysis identified three intersecting themes: (1) navigating complex, adult systems; (2) distress, grief and trauma; (3) acceptance, normalisation and coping mechanisms. In this paper, we illustrate how these themes were harnessed to co‐produce a child‐centred framework for social visits based on children and young people's priorities for change, a framework we articulate as ‘The Three Cs’.

**Conclusion:**

Contact, which strengthens family ties and protects health and wellbeing, requires approaches which are child‐centred, consistent and compassionate. Crucial to this are enhancements to prison officer mandatory training on family ties and the impact of family imprisonment as well as exploration of how to harness existing support pathways designed for vulnerable children and young people to ensure that those experiencing familial justice‐involvement do not fall through gaps in service provision.

**Patient or Public Contribution:**

From its inception, this project was a partnership between academia and two voluntary sector organisations that support families experiencing imprisonment with the core project team being split between academic and practice‐based partners (reflected in this article's authorship). An international stakeholder group was also convened to support the study across its duration and who supported the research team in the development of research questions, topic guides and participant materials, guided the interpretation of our findings, and provided input into our impact and dissemination strategy. This group met quarterly and included representation from voluntary sector and grassroots organisations, academics with experience of working with children, families and justice‐involved populations, prison and probation service colleagues and creative practitioners. Aligned with this partnership approach, we established regular satellite check‐ins with other voluntary sector organisations across the United Kingdom who support families experiencing imprisonment to embed relationality and feedback loops for actionable change. Five study participants were involved in the co‐creation of comics, illustrated by Jack Brougham (see ‘Materials and methods’ section for further insight). Finally, during the analysis, dissemination and impact phases of the project, we worked with a youth board of children with experience of familial imprisonment (recruited through a partner organisation) to sense‐check our findings and to develop a campaign video (an approach chosen by the board). The video was based on the analysis of project data and the youth board's own experiences. This process was held over 3 full‐day sessions, co‐facilitated by a locally based arts organisation and involved a range of creative activities. Participants were thanked for their time with gift vouchers. Both processes with young people (data collection and engagement activity) further informed our analysis and were fundamental to shaping our three Cs framework, a framework co‐produced with our core voluntary sector partners and illustrated by Nifty Fox.

## 1 Introduction

The Ministry of Justice (MoJ) reports that there were 192,912 children with a parent in prison in England and Wales between 1 October 2021 and 1 October 2022 [1, p. 2]. However, this figure remains a conservative, indicative and modelled estimate only, as those entering custody do not always declare dependants. Also, this figure does not account for non‐parent family members, such as siblings, grandparents, etc., as well as chosen family; prevalence data which—to date— remains unavailable. We also know very little about the population‐level health and wellbeing impacts of having a family member in prison upon children and young people in England and Wales and how this experience links to other detrimental experiences either in tandem or in later life. What we do know from broader, international extant literature, is that children who experience familial imprisonment appear to be at greater risk of disrupted attachments, economic instability, disturbed educational pathways, substance use disorders and poorer mental and physical health outcomes than their peers both in childhood and later adulthood, worsened by experiences of stigma and social isolation [2, 3, 4, 5, 6, 7, 8, 9, 10, 11, 12, 13, 14]. Indeed, the imprisonment of a household family member is one of ten recognised adverse childhood experiences (ACE), defined as exposures to severe stressors such as emotional, physical, and sexual abuse as well as growing up in challenging home environments [15]. ACEs tend to cluster and compound each other, with poverty recognised as a key driver [16]. In other words, the more ACEs a child experiences, the more likely they are to experience a myriad of adverse physical and mental health outcomes which can extend into adulthood [17]. Thus, imprisonment of a household member is associated with a fivefold increase in exposure to other ACEs [18]. For many children impacted by familial imprisonment, adverse outcomes are further exacerbated by other marginalising factors such as poverty and disadvantage, and systemic racism, sexism, homophobia, transphobia, and ableism [11].

Promisingly, at the time of writing, the current UK national government's election manifesto included a clear commitment to supporting children affected by parental imprisonment [19]. However, it remains unclear what direction this support will take and where statutory responsibility ultimately lies to support children impacted by family imprisonment, with accountability for this group of children seemingly dispersed between the MOJ, the Department of Education (DfE) and the Department of Health and Social Care [13]. In other words, without clear recognition of where this responsibility lies—and a clear statutory duty to report accurate data as to who families experiencing imprisonment are—this group of children is likely to remain unnoticed and unsupported [13]. Crucial to this policy is how such intelligence is ‘used’, with concern mounting as to whether the identification of families experiencing imprisonment could result in scrutiny rather than support; as well as further pontification of ‘intergenerational offending’, whereby children with a parent or parents who offend, are thought to go on to offend themselves—a phenomenon contested widely as reductionist in scope and incredibly stigmatising [20, 21]. Meanwhile, whilst the MOJ has a duty to strengthen family ties, their responsibility for this relates to the reduction of re‐offending only. Instead, it is the DfE and the Department of Health and Social Care who are expected to support children and families more broadly. In comparison, as of 2024, in Scotland the UN Convention on the Rights of the Child (UNCRC) is incorporated into domestic law, meaning legal challenge is possible where children's rights are breached (though the implementation of this law into practice is ongoing). Ultimately, we suggest here that whilst investment in families of people in prison is to be welcomed, where government policy situates the rights of children experiencing family imprisonment in the context of offending behaviour only, rather than by focusing on their right to thrive and to a family life, both system change and improvements to health and wellbeing are likely to remain limited.

A growing body of sociological and criminological research explores the impact of imprisonment on families (such as [3, 5, 9, 10, 11, 12, 13, 22]). However, to our knowledge, very few studies have centralised the voices of children and young people in their work. Therefore, a few notable exceptions withstanding, extant data are predominantly derived from adult family members who focus on their own lived experience or who act as a ‘proxy’ voice for their children; curated from small‐scale evaluation of specific community‐based support and/or interventions; or from studies where children and young people are involved as participants alongside adults. To our knowledge, no previous studies have spoken to children and young people over a prolonged period, and none have focused explicitly upon health and wellbeing. Our intention here was to go some way towards evidencing this gap. Drawing on an ESRC‐funded project encompassing serial longitudinal interviews and the curation of creative methods with 19 children and young people (age 7–16) who have a family member in prison across Northern England and Scotland, the aim of this research was to explore the impact of familial imprisonment on children and young people's health and wellbeing, and utilise our findings to develop a rights‐based framework for prison social visits. In doing so, this constitutes the first major research project across England, Wales and Scotland to speak directly to children and young people experiencing family imprisonment about health, wellbeing and inequity.

In the first section of this article, we briefly unpack what is known from extant literature about the ‘pains’ of familial imprisonment, focusing specifically upon symbiotic harm, stigma, loss and grief, concepts we expand upon in a forthcoming paper delving deeper into children and young people's longitudinal narratives of family imprisonment. In the second part, we outlay our study context (the ‘Divided Households’ study) and our overarching findings from this piece of qualitative longitudinal research. Third, we present how these findings were used to underpin a child‐centred framework for social visits based on children and young people's priorities for change, a framework we articulate as ‘The Three Cs’ [23]. Finally, we reflect critically upon this framework to examine the current fragilities in health, care and justice ‘systems’ and the potential utility in ‘whole system’ approaches which aim to ensure that those experiencing familial justice‐involvement do not fall through gaps in service provision.

## 2 The ‘Long Shadow’ of Family Imprisonment

A small but burgeoning body of literature has used a myriad of terms to describe the harms of imprisonment upon imprisoned people and those close to them, much of which originates from Sykes [24] notion of the ‘pains’ of imprisonment. For family units, authors have tended to conceptualise this as an extension of the sentence that the person in prison has received: i.e. a ‘hidden’, ‘parallel’ or ‘secondary’ sentence [1, 5, 25, 26]. Such secondary harm has also been described as ‘symbiotic’ by Condry and Minson [5] to illustrate the complexity and enormity of the impact that imprisonment has upon families. They conceptualise ‘symbiotic harms’ through combining literature on the ‘collateral consequences’, which describes the negative impacts of imprisonment that go beyond a custodial sentence on the individual, and literature which addresses the impact of imprisonment on families to address the complex and multi‐dimensional negative effects of imprisonment on people in prison and their families. The concept of symbiosis reflects how such harms ‘flow both ways’ within individual, collective and institutional relationships in complex, fluid, far‐reaching ways. Therefore, ‘symbiotic harms’ underscores how the effects of imprisonment extend far beyond the individual, impacting families and communities interdependently. Thus, for Condry & Minson, symbiotic harms *‘threaten social justice, human rights, and the democratic organisation of society and ought to be central to any endeavour to theorise the social ramifications of prison and punishment’* [2020: p. 18].

One such parallel ‘harm’ is the removal of a family member from most aspects of daily life, likened in previous literature to the loss experienced as a result of bereavement. In the context of imprisonment, the concept of ‘ambiguous’ loss or grief—originally applied to exploration of the emotional impact of paternal absence ‐ has been used to articulate the complexities of loss where the ‘usual’ coping mechanisms and responses to grief are interrupted, disrupted or blocked [1, 27, 28]. Grief that results from such ambiguous or disenfranchised loss differs in that it tends to be liminal, lacking certainty and closure for families [27, 28]. Similar to the loss and grief of bereavement, the loss of a parent to imprisonment can have a demonstrable impact upon children and young people's physical and emotional health and wellbeing, with symptoms of post‐traumatic stress common for both types of loss [29, 30]. Yet, unlike bereavement, the family member is still alive but physically and emotionally less/unavailable, making the processing of the loss more complex. Meanwhile, the lack of public recognition or support for this kind of loss can deepen feelings of isolation and sadness. As ambiguous loss is associated with feelings of ambivalence, helplessness, identity confusion, insecure attachment, and hopelessness [28, 31], it is our assertion here that this form of loss can have a profound impact on the health and wellbeing of children with a family member in prison, and that appropriate, child‐centred contact with family members can mitigate some of this impact.

Thus, where permitted and appropriate, social visits and family contact represent a vital protective factor for the mental health and wellbeing of those in prison as well as their families and significant others [32, 33, 34, 35]. Flynn et al. [32], for example, demonstrated how family bonds and connectedness are core to child wellbeing, illustrating how visiting can help to mitigate the negative impacts of imprisonment of fathers. Nevertheless, prison visiting and maintaining regular contact come beset with practical and emotional challenges for families and loved ones, such as restrictive visiting times, lack of privacy and/or suitable and safe play spaces for children, geographic distance and expensive/poor public transport options [33, 36, 37]. The recent ‘Social Contact in Prison’ report published by the MoJ [38] highlights that social visiting numbers dropped during the pandemic due to restrictions but have not yet returned to levels of visiting prior to the pandemic outbreak. The report also acknowledges barriers to maintaining contact, particularly the cost of visiting and phone calls, and inconsistent options for contact depending on the prison site, as well as regional differences in amount of visitors that prison populations receive (assumed due to cost and distance, though more research is needed).

Visits may also create or exacerbate tension or strain in certain relationships [39, 40], whilst others have recognised that families visiting prison can be viewed as a security threat rather than as a source of support for their family member [41]. Meanwhile, tabled amendments to the Victims and Courts Bill [42], outline important moves to restrict the parental rights and responsibilities of parents convicted of serious sex offences. Therefore, whilst beyond the remit of the study explored here, it remains imperative also to understand—as a counter‐narrative—what happens when loss through family imprisonment cannot be mitigated using contact and prison visiting, and how children and families can be best supported where contact is not permitted, wanted or in the best interests of the child.

## 3 Materials & Methods

The project upon which this article is based is qualitative and longitudinal by design, comprising serial interviews with children and young people aged 7–16. The project took place between May 2022 and March 2025 and represents over 45 h of interviews. During this time, 19 children and young people (10 girls, 9 boys) agreed to take part in up to three one‐to‐one interviews (held roughly every 3–4 months). All young people interviewed had a family member currently serving a custodial sentence in a prison in Northern England or Scotland at the time of recruitment. Collecting data across two geographical sites in this way was intended to allow exploration of two different but comparable legal systems (‘England and Wales’ and ‘Scotland’). Recruitment predominantly took place in Prison Visitors' Centres. In Scotland, recruitment was supported by Family Support Coordinators employed by one of our partner organisations. We did a number of things to centralise the wellbeing of those we interacted with during recruitment, working with our partner organisations and their expertise. Firstly, based on discussions with our partner organisations, we did not conduct any recruitment within remand prisons due to the intensity of experience at the early stages of familial imprisonment. Many children and families may have been visiting for the first time, and we did not want to cause overwhelm. We also worked with staff in the visitors' centres, who often had relationships with those who visited regularly, before approaching families to participate. Meanwhile, we were mindful of the potential that some children may not have been aware that they were visiting a prison and took care in how we approached families to discuss the research and in the language that we used.

All interviews were conducted by one researcher (NG) to build continuity and strengthen rapport. Interviews took place in family homes and public spaces such as coffee shops (based on participant choice) to ensure comfort for participants. Three participants opted to take part in a mixture of online and in‐person interviews (at their request, based on geographical distance and difficulty scheduling), but all participants who took part in an online interview also took part in in‐person interviews. Given the potentially distressing nature of the topics we covered, we were careful to build in regular breaks and check‐ins during the interviews. Participants were also given the choice to have a family member or friend present at the interview, either in the room or nearby (usually in the next room). To address power imbalances that are inherent in research conducted between adults and children, the obtaining of fully informed consent was vital [43], therefore we created an accessible child‐friendly information video with illustrations and music to help to ensure that participants understood their rights. As interviews took place over multiple sessions, we sought written informed consent during interview 1 but re‐established consent verbally at the beginning of each subsequent interview, particularly reasserting the opportunities for pausing, stopping and withdrawing throughout at any time.

**Figure 1:**
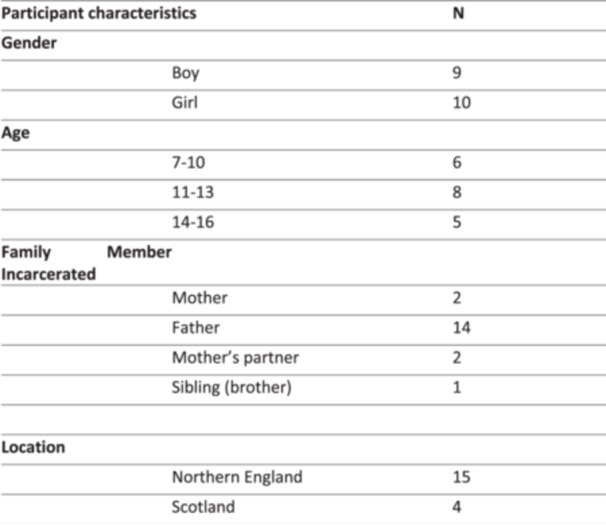
Participant characteristics table.

Methodological reflections, strengths, pitfalls and nuances are prudent to reflect upon here. First, it was made clear to potential participants during recruitment that a ‘family member’ could be anyone whom they considered to be family. Family members represented were therefore fathers, step‐fathers/mother's partners, one sibling and one mother (of two participants). Figure 1 below provides a detailed visual breakdown of our study sample. Second, recruitment was exceptionally time‐consuming and warranted significant resources. Thus, whilst our sample is diverse in age and gender, it is tempered by the vast majority of our participants being white (*n* = 18).

Meanwhile, despite our aim to have a more even split across Northern England and Scotland, the majority of our participants were in the North of England (*n* = 15), we believe due to proximity to the research team and visitor centre access and size. Finally, the vast majority of participants’ imprisoned family members were men (*n* = 17). Whilst we cannot attribute this with any degree of certainty, we believe there are two key factors in this difference in sample—the main factor being that we were granted greater access to prisons in the male estate. However, we also perceived a greater reluctancy generally to take part from families visiting women in prisons. We are aware of the particular challenges maternal imprisonment brings in relation to social care involvement and how this shifts family dynamics and vulnerabilities for children experiencing the imprisonment of their mother, with only 5% of children whose mother goes to prison able to stay living in their family home [44, 45, 46]. Given our commitment to a feminist ethics of care, this reluctancy is something we wish both to respect and acknowledge. Moreover, this speaks to the challenges of ‘research labour’ (both practically and emotionally) in this space tending to be orchestrated by women (both as researchers and participants). In other words, the fact that most of our sample were experiencing the imprisonment of a male family member may be an artefact of the fact that during data collection, it tended to be mothers who were more open to the prospect of taking part in a research study and positive about the impact taking part would have on their child. Further, it is also important to point out here that—as most of the prison population in England, Wales and Scotland are male—t of course stands to reason that this will also be the experience of most of our interviewees. Finally—and importantly—aligning with our points above relating to children and families who do not visit either through choice or factors such as distance or court orders [47], the participants in our research comprise children and young people who have,^1^ and who want to visit.

All three interviews incorporated visual, creative activities to help participants (some of whom were quite young) to feel comfortable to facilitate participants sharing their stories. Leitch's [48] work illustrates how creative methods can help children to both express and to understand their feelings, especially where those feelings may be complex. We endeavoured to offer activities that were appropriate for all of our participants, that were interesting and engaging while allowing participants to go into as much or as little personal depth as they felt comfortable [43]. The creative activities conducted in this research also acted as a vehicle through which the researcher and the participant could connect and build understanding [49]. The first interview focused on the practicalities of visiting a family member in prison. During this interview, children and young people completed a mapping exercise where they drew a map of their visit, and we talked through their thoughts and feelings at different stages of the visit. The first session was deliberately high‐level and allowed participants to get to know the researcher, so the session was designed to be quite practical and procedural in focus. To help with rapport building and getting to know each other, the researcher also asked each participant what their favourite snacks were, then brought them along to the following sessions. The second interview focused more on the children themselves and their wellbeing. As part of this, we carried out an activity called ‘worry mapping’, drawing from literature on discussing wellbeing with children and young people, as well as experience and expertise from the project team. We talked through different aspects of life and mapped them onto a ‘worry table’ from ‘never’ to ‘all the time’. This was a way to get to know the participants better and to see how they felt day‐to‐day and the impact of stressors on their wellbeing, a depth which we felt would be best placed in the second session, where familiarity and rapport had had time to develop. The third and final interview focused on children and young people's priorities for change and combined learning from the first two sessions for each participant, with conversation prompts created for each individual based on previous conversations. The activity in this session was character creation, where the participant created a child or young person who was experiencing familial imprisonment and explored the impact through narrative storytelling. The participants chose all of the characteristics of the child in question, and their responses varied in how closely their character matched their own experience. We felt this approach would allow some distance from the difficult subject matter for participants, allowing participants to choose how much of themselves they reflected in their character. We also wanted the 3rd session to focus from the more in‐depth personal focus of the second session to broader themes around changes that they would like to see within and beyond the criminal justice system.

After each interview, the researcher would check in with the participant or a family member where direct contact was not possible (particularly with younger children), to see that they were OK after the session and offered the opportunity for feedback for future sessions. Responses and feedback were overwhelmingly positive. At the end of the last session, participants were also given a list of resources for further support, including options for engaging with our partner organisations who offer tailored support in the recruitment areas, as well as wider resources to support mental health and wellbeing.

We also worked with a smaller subset of children and young people who took part in our study and a professional sketch artist (Jack Brougham) to co‐create comics based on the character creation activity carried out in their 3rd data collection session. To maintain anonymity, the participants met with the project researcher to discuss comic plans and drafts at each stage then subsequently relayed participant feedback to the artist. This process was repeated until participants were happy with the comics. These sketches also formed the backbone of our Three Cs framework ‐ a framework co‐produced with our core voluntary sector partners, illustrated by Nifty Fox, and which we outline in the manuscript sections that follow.

Ethical approval for this study was provided by [23] (Reference: 2346/23630). All children and young people received a £20 voucher at the end of each interview to reimburse them for their time. All interviews were digitally recorded, transcribed and organised using both Microsoft Excel and Nvivo. We took a reflexive approach to thematic analysis to ensure that a collaborative coding approach was used to analyse the data [50]. All transcripts were double‐coded, first by NG, and with SS and LC splitting the role of second coder between them. We held weekly analysis meetings to continually revisit and reinterpret, using Miroboards to document our discussions, observations and analytical insights. Written notes were also maintained during data collection in the form of a reflexive research diary. This had the dual purpose of allowing the researcher to reflect on the research process and their own positionality (and that of the team) during data collection, and provided further context that we could draw upon during later analysis to aid interpretation of the data. Project meetings were also held regularly with the core team, where practical project planning was discussed as well as ethical concerns, participant and researcher comfort and safety, and positionality were discussed in an open and supportive environment. As a research team, we have no direct lived experience of the justice system, but the lead author also has a wealth of experience working with children and young people affected by marginalisation and disadvantage in social research, educational and youth work contexts. Also, as authors, we take a dual role as researchers and advocates for a more equitable justice system in Scotland, England and Wales. This is also demonstrated in our authorship team which is a partnership between academics and community organisations that support people involved in the justice system and their families.

To ensure researcher safety during data collection, we had a buddy system where check‐ins were scheduled before, during and after interviews had taken place. To support researcher wellbeing there were regular check‐ins with their line manager and project lead (weekly) as well as ad hoc check‐ins around more intensive periods of data collection. The lead author also had a mentor for additional academic and wellbeing support.

Managing the emotional burden of having a family member in prison meant that children and young people in our study experienced grief, relief, trauma, shock, confusion, stress, worry, problems at school, changes in their ability to focus, impacts on their relationships with others, and changes to their sleep quantity and quality. Our reflexive approach to analysis resulted in three interconnected themes that we felt were salient to children and young people's stories: (1) navigation of complex, adult systems; (2) distress, grief and trauma; and (3) acceptance, normalisation and coping mechanisms. Our focus in this article is children and young people's interactions with prison sites, their suggestions for system change and how these insights informed the development of the ‘Three Cs’ Framework. Therefore, whilst these three themes underpin each component of the ‘Three Cs’ Framework that follows, longitudinal data from this study focusing on the emotional impact of familial imprisonment upon children and young people in greater depth will be published separately elsewhere in a forthcoming paper to afford it the space it deserves.

## 4 Results: Young People's Priorities for Change: Developing The ‘Three Cs’

**Figure 2:**
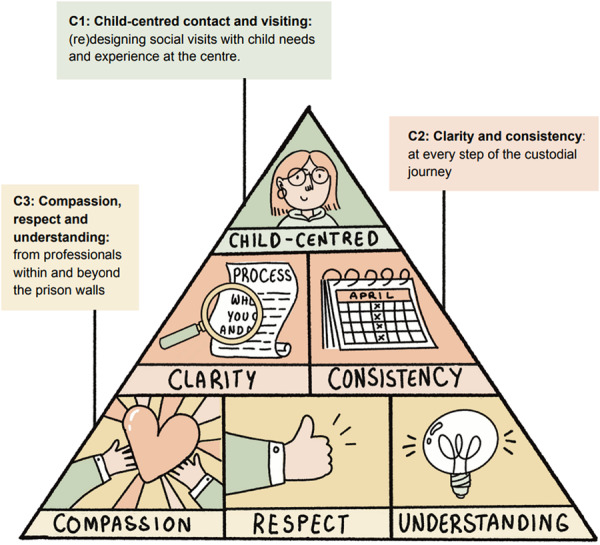
The Three Cs framework. Illustration created by Nifty Fox for the project report [23].

Building on the themes outlined above, and in dialogue with our voluntary sector partners and children and young people who participated in our project, our Three Cs Framework [23] identifies interconnected and cross‐cutting priorities for change in relation to prison visits and family ties, with each ‘C’ intentionally signalling to the differing levels of change required to improve the health and wellbeing of children and young people affected by familial imprisonment (see Figure 2 below). Thus, ‘C1’ focuses on the need to (re)design prison visiting and family contact with child needs and experience at the centre, which we believe, in turn, will benefit all visitors. Many of the suggestions related to C1 are fairly straightforward and even occur in some of the prison sites covered by the study, but are inconsistent. ‘C2’ expands beyond ways to maintain contact with a loved one to the need for structural, cultural and practical change to provide clarity and consistency at every step of the custodial journey, from arrest to release, to reduce the negative impact that familial imprisonment can have on children and young people. Meanwhile, ‘C3’ expands beyond, but still includes the criminal justice system to call for compassion, respect and understanding from all professionals who engage with children and young people. It is often not possible to tell which children are affected by familial imprisonment due to concealment as a result of fear of judgement. We therefore call for greater awareness and training of professionals who work with children and young people within and beyond the prison context, pushing for greater investment in supporting children and families affected by the criminal justice system across sectors. Here, we expand upon and unpack each ‘C’ in turn, using findings, quotes and visuals developed during our research to contextualise our framework. We use pseudonyms throughout; no characteristics are attributed to quotations used to safeguard the anonymity of our participants.

*‘That's what helped me. And I spoke to him, like, every night. And him telling me, “It'll be all right”*

### 4.1 C1: Child‐Centred Contact and Visits

#### 4.1.1 The Importance of Visiting

Participants described the emotional strain caused by uncertainty and the sense of loss associated with having a family member in prison at length. They discussed shifts in family dynamics, separation and divorce, having to move house and the need to support their other family members (usually their mum and siblings) emotionally ‘beyond the prison wall’.

…well, before he went in it was just every day, ‘Howay, shall we go out somewhere?’ So they were used to just going places and that with him all the time. And then just all of a sudden it went…

Frankie

Access to regular contact and visits mitigated against the stresses and strain they faced and provided a form of reassurance, often driven by welfare concerns about their imprisoned relative's wellbeing, particularly at the beginning of a custodial sentence. Knowing that their family member was safe allowed participants to feel more at ease and to ‘relax’ in their daily lives.

Visits were valued inordinately for the opportunity to spend time together as a family. This time was deeply meaningful but often complicated by institutional restrictions. For example, limits on the number of visitors meant that, for some participants, not all siblings could attend visits simultaneously. As one participant shared:

Best thing's just being able to spend time with my brothers and my dad together.

Frankie

The act of sitting and talking as a family created moments of connection where the prison setting faded into the background, offering much needed emotional relief amid the broader challenges of familial imprisonment. One participant described this feeling, saying:

Do you know, when you're there you forget where you actually are, you just feel like you're sat speaking and then you just you zone back in. Even my dad says, ‘Oh, I forgot I was in here for 5 min.

Frankie

Physical contact, though also limited, was an important part of these interactions, helping to maintain a sense of closeness and familial connection. For some, in‐person visits also offered a temporary return to a sense of normality.

Well, I suppose I actually get to see him, and I like playing games with him… I like that you can actually‐ not actually touch him, but you can give him a hug.

Sarah

The above quote shows the importance of physical contact while illustrating that this is restricted in most visits.

#### 4.1.2 The Cost of Visiting

Children and young people in our study identified numerous ways that prison contact and visiting could be made more appropriate for them and centralise their needs. The most commonly raised concern was related to the infrequency of visits, particularly family visits, and the difficulty in securing a place due to high demand. However, whilst children consistently expressed a desire for more frequent access to visits, recognising their unique role in maintaining meaningful family connections during imprisonment, there were many aspects of prison visiting that participants found difficult. This included noise, a lot of time waiting, inconvenient visiting times (often clashing with school), lack of privacy, adult language, and the distance they needed to travel.

The financial cost of visiting was raised by the majority of participants in some form, linked to travel as well as the cost of food and drink once in visits, and lost earnings through limited options for visiting times.

[I visit] Just when I can afford it really. Because the time we get there and then it just costs a lot. I try and get in like once a month, if not like a few times. Just depends.

Mother of Sydney

The cost of contact also extending to phone calls, which were vital for many participants' sense of closeness and routine.

*Interviewer: How often do you talk on the phone?*
*Brianna: Every day. Or if he doesn't have enough credit, every few days*.

The costs of visiting must also be viewed in the context of broader costs (including loss/reduction of household earnings) as a result of imprisonment:

They hardly get owt to eat. They don't get money. What's it? £1 or £2 a day for working in there. It's left to us to try and fork all this money and to keep going as well.

Dean

In relation to food, participants welcomed the opportunity to eat with their family member in prison, but generally complained that the food options were limited and were mostly snack foods and were not cheap.

But it's just mostly chocolate and that [and] it's so expensive.

Dean

Several participants commented on the reduction in quality and options for food as a result of the pandemic, then remaining limited even when other aspects of visits had resumed. For Omar, there used to be a cafe accessible within the hall that he really missed, as he could have a meal with his father, rather than vending machine snacks:

But since COVID, they've not been back. I don't think they're coming back… Bring back my kitchen!

Omar

#### 4.1.3 Processes and Staff Attitudes

In particular, children and young people faced anxiety and stress as a result of the security process, particularly on their first visit. Where participants had had someone from the visitors' centre (generally run by voluntary sector organisations in our sample) go through the visiting process with them in advance, anxiety was greatly alleviated.

I think they [younger brothers’ were more just scared, because they'd heard… Well, everyone hears bad things about them [prisons], and they just thought prison was just a really dangerous place.

Frankie

However, not all children had this opportunity. Further, whilst video walk‐throughs could also provide a helpful way to show people what to expect on a visit, participants involved in this study had not had access to them. Most participants found that they became more used to the search process after a few visits and were more at ease, seeing a general move from feeling scared and anxious to acceptance after several visits. However, participants experienced several common frustrations, many of which centred on the attitudes and actions of prison officers:

Half of the screws and everything here are really moody, stuck up.

Hannah

Some participants found certain prison officers to be friendly and humorous, while others encountered officers who were strict and unapproachable:

Some of them are nice. One of these on here is horrible. Like really horrible… Then you can get really nice ones, and then you can get proper moody and horrible ones. So, it all depends who's on really.

Kelsey

The demeanour of the officers notably influenced the children's experiences of being searched. Participants appreciated when officers were friendly, calm and reassuring. Many felt more at ease when they were allowed to interact with search dogs in a relaxed manner before the search. However, this practice was not consistently available across different sites, an observation especially relevant for those with experience of visiting multiple prisons, where the variation in staff behaviour could significantly affect the overall experience.

If I'm being honest, I hate half of them [prison officers]

Omar

Participants appreciated when officers were friendly, calm and reassuring. Many felt more at ease when they were allowed to interact with search dogs in a relaxed manner before the search. However, this practice was not consistently available across different sites, an observation especially relevant for those with experience of visiting multiple prisons, where the variation in staff behaviour could significantly affect the overall experience.

But [Staff member] who worked there, he always let us see the dogs and everything. So, everybody went in and I'd have a couple of minutes with the dogs. I would put them in their cages and everything, and help with them. Whereas, this one [another prison], they pat you down and everything, and they just set the‐ They try and get rid of you quickly.

Kelsey

### 4.2 Visuals & Messaging

While family visits were overwhelmingly preferred by participants to regular social visits, the context for those visits was the same—the visiting hall. Participants described visiting halls as plain with nothing to look at and often complained that the chairs were uncomfortable and unable to be moved. Visiting halls felt unwelcoming, and one participant even described how their visiting hall used to be brighter and more colourful but that they painted the walls:

They used to have like designs on the walls and that, but they've painted over them now.

Omar

The majority of the participants also complained that there is generally very little to do on visits, with, at most, a pen and paper provided. They particularly reflected that there was very little for older children to do, as play areas, where available, were for young children.

Here is the paper, but we've got no pens.

Kelsey

Some participants were frustrated that they could not take anything in with them for security reasons (e.g., one participant would have felt better if she could take a soft toy into the visit with her and others discussed wanting to show their family member things they had drawn).

Participants also vocalised acute awareness of people in prison not being allowed to wear their own clothes, and that some people in prison wear a different colour to represent them being in solitary confinement ‐ a visual representation for all in the hall that that person was being punished further. *‘So my dad comes in, in his red top, and then whatever bottoms he wants. Everyone has like, a red top. And then if you're in like solitary, you need to wear a grey top…So, makes you look like an odd one out if you're in solitary’ Omar*.

Participants also highlighted the discomfort and lack of privacy associated with the presence of numerous prison officers and the proximity of other tables, which allowed visitors to overhear other conversations.

Because I hear some of that conversation sometimes so they might hear some of ours.

Gareth

These factors contributed to the perception of a tense and unwelcoming environment within the visiting halls. In contrast, visitor centres, often operated by charitable or third‐sector organisations, were generally described as more vibrant and colourful, with toys and books for children to engage with while waiting.

#### 4.2.1 Family/Children's and Other Visits

For the reasons highlighted, ‘family’ or ‘children's’ visits were deemed best, with visits in open prisons preferred even more. These visits differ significantly from standard social visits in both structure and atmosphere. While the provision of family/children's visits varies between sites— they tended to be described as more relaxed, allowing greater freedom of movement for both children and the imprisoned family member, and offering a wider range of activities.

It was much better… like, a game night really, but obviously in the morning.

Kelsey

Those who had the opportunity to attend consistently expressed a strong preference for them over regular visits. They particularly valued the increased opportunities for physical contact, the ability to engage in play, and the overall sense of normality these visits provided. Participants also highlighted the emotional significance of being able to take family photographs during these visits:

I mean last time they took photos which they'd never done before… they came back with three, two for us and one for him… I know he's got photos of us three together but I don't think he had a photo of all four of us together.

Kelsey

Such photos were important both as personal keepsakes and as a way to maintain a sense of familial identity. One participant, who had chosen not to disclose their parent's imprisonment to friends, noted that having a recent photograph with her father would be helpful when asked about him. However, not all participants had been to a family or children's visit:

What's that?… Don't even know what it is.

Dean

In the same vein, whilst ‘virtual visits’ (video calls) were welcomed, they could be glitchy, inconsistent, and there was a strong consensus that they do not and should not replace face‐to‐face contact Yet virtual visits^2^ offer a necessary alternative when in‐person visits are not possible (e.g., due to availability of family visits, distance, accessibility, and in the case of individuals who may struggle with the prison visiting environment as it stands, such as neurodivergent visitors).

…they change the rules as they go as well. Some of them don't know the rules.

Omar

### 4.3 C2: Clarity & Consistency

#### 4.3.1 Differences between Sites

Children and young people with a family member in prison were expected to navigate and understand a complex justice system designed for adults. It meant that—with the exception of quite small children—they were expected to behave as an adult would within prison settings but lacked power, agency or control over their circumstances. Further, the prison system children and young people found themselves within was unclear and inconsistent, heightening the stress and anxiety we describe above in C1. Many of our participants had experience of visiting multiple prison sites, and they referenced differences between these sites in terms of provision, visiting processes and rules:

[At the last prison] you could go whenever you want. You could speak or visit‐ Because you could go‐ If you book Saturday and Sunday, you could go there, but this one you can't. You're only allowed two [a month].

Kelsey

To our participants, these differences were confusing, frustrating and arbitrary. Such differences included, but were not limited to: different rules about clothing and jewellery (including whether you have to remove jewellery when being searched), varying rules about the number of visitors, how much and what type of food you could buy for the visit, and what level of physical contact was permitted.

But in the old one, I didn't need to take my birth certificate or anything. Then obviously, I would just go and see my dad whilst they get their fingerprints done. Whereas here, I've got to wait.

Kelsey

Participants also described different provisions in different sites, such as different times of visits (which may be more or less convenient in relation to school and public transport), different types of visits available, and differences in frequency and capacity of family days. For example, one participant described a father and child homework club at one prison that was not available when he was moved to another prison. The time which the young person spent doing homework with her father was important to the young person, and she really missed it when her father moved prisons.

### 4.4 Changing and Inconsistent Rules

Multiple accounts were shared describing being given permission for something by one prison officer then being reprimanded for it by another, causing great distress and adding to a lack of trust between children and prison staff. One participant, age 12, also described arbitrary rules around physical contact where she was reprimanded for hugging her father as prison staff had assumed she was over 14 (where the cut‐off was) causing her great distress:

And then there was that point as well where it was after COVID, it was if you're under 14 or 14 under, you were allowed to hug your dad and that. So I gave my dad a hug and then they all went mental. Like, “Oh, you're not allowed to do that.” But we had been told before that I was allowed to and people up there said that I wasn't. There was a big thing about that.

Hannah

This resulted in children and young people feeling continually punished by staff who made the rules for the actions of their adult family members. It also resulted in a lack of relationality and trust between staff and families.

But there'll be like a week where if it's one member of staff, I can get in with my hood, but as soon as I get upstairs and there's another one, they say, “No, you can't have that one.” … So, they're not all on the same level.

Omar

Participants were also expected to understand and accept inconsistencies in the length of visits, as well as last minute cancellations on some occasions without warning. Several participants described frustration when they had been on time for a visit but had had to wait so long for their family member to enter the hall that their visits were reduced significantly:

When we first went, I think we had not even half an hour. By the time you get over and then the time that he‐ he was literally the last one out and he had no more than half an hour. That was hard, that, like, God, just think about it‐ it broke her heart.

Mother of Sydney

Along with the financial strain and the requirement to travel long distances to visit a family member discussed in C1, sudden and often unanticipated movement of family member caused an additional hurdle or change to continually adapt to for children and young people impacted by imprisonment.

I think I've been to see Dad three times since then. But I don't like how far away it is, so I don't go as much as [Sister] and my mam… Because I can't sit in the car for too long. I cannot sit that long. It stresses me out.

Hannah

Participants described frustrations and stress when family members were moved without warning, especially when moved further away, with two participants having to travel approximately 4 h each way for their visits.

It was in the middle of nowhere.

Noah

#### 4.4.1 The Stress of Uncertainty and Confusion

Central to young people's stories were high levels of uncertainty (about release, movement, and safety of their family member); and this uncertainty impacted upon every aspect of their lives. other participants described uncertainty and a lack of clarity around the often‐changing timelines in terms of when family members could first speak on the phone, first have visits, the sometimes sudden and unexpected movement of a family member between prisons, and a lack of clarity around when they would be released. Several participants had expected their family member to be released for several months before the release actually happened, which caused dashed hopes, frustration and stress. Mabel was uncertain about how long her father would be in prison for the whole length of the project, with her saying in each interview that she hoped he would be home by the time we next would meet. By the final interview her 13th birthday had passed, and she was then certain he would be released in time for her 16th birthday. Her expectations changed in each interview, using her birthday as a milestone for when she would be able to see him and being forced to accept the changing timelines that were out of her control:

Interviewer: [You said] last time you wanted him to be out for your 13th, so that is hard.

Mabel: Yeah, but the only good thing is he definitely will be out for my 16th.

Thus, ‘acceptance’ as a means of coping for the children and young people in our study included lowering their expectations about prison visits, wider professionals and from their family members —perhaps, ultimately, to protect themselves from being let down. One participant described his younger brothers' choice to have the release of their father remain a surprise, to avoid disappointment. Their father was initially given a 6‐week sentence but remained in custody for 17 months:

One of the first things he [younger brother] said was, “Can you not tell me when you're getting out?” He didn't want to know…. because he's only young, he might have not wanted to know in case it was going to be a while.

Frankie

Importantly, lack of clarity and consistency were not simply associated with children and young people's experiences of visiting prison. Stress, shock and confusion tended to begin at the point of the arrest of their family member, particularly where participants were present when the arrest happened. One participant described intense stress on the day of arrest when the police arrived at their home, with no one explaining what was happening or where their family member was going. Another described a regular occurrence of being sent to a family member's house nearby while the police raided their house, but not understanding what was happening:

I was obviously upset and a bit scared. So what kind of happened is some people came to the house and I just had to pack some clothes and stuff and then I went to go live with my aunt for a couple of weeks, because my mum had to go in to do some stuff to do with what happened.

Sarah

So, in my primary, if my grandma was picking me up, they would all just look at her after it happened. Because they would know.

Kelsey

### 4.5 C3: Compassion, Respect and Understanding Within and Beyond the Prison Walls

#### 4.5.1 The Emotional Impact of Imprisonment on Children

Connecting C1 and C2, we suggest that experiencing a family member going to prison can have a profound impact on the lives of children and young people. For our participants, it impacted their education, relationships, health and emotional wellbeing.

Children are required to cope with changes to family dynamics and for children who leave their home as a result of familial imprisonment, changes to their entire living space as well as routines and structure, and for some even changing schools.

…my mum and dad ended up splitting up… And they've been together for 14 years…

Omar

The children and young people in our study experienced grief, relief, trauma, shock, confusion, stress, worry, problems at school, changes in their ability to focus, impacts on their relationships with others, and changes to their sleep quantity and quality. We contend here that such narratives —and young people's experiences of loss can reflect the stages of grief, with some children and young people eventually landing on ‘acceptance’:

I wouldn't speak to anyone, for the first month that I knew he was in jail… I did not sleep. I didn't go to school for a whole month… Like, I would not go to sleep. And if I tried to, I could not. Like, just knowing about it, I just couldn't. Then one time I went to sleep, and one time I woke up, and I just thought, “Just get over it. It's been a whole month.

Noah

To do so, they appeared to enact their own coping mechanisms to assert a level of control over an otherwise unpredictable situation. With Tia giving the following advice to children in her position:

Probably just, like, try avoid thinking about them, because it might make you sad.

Tia

For Kelsey, watching TV helped her to cope, by escaping into another world for a while:

You're just more interested in the series and you want to know what's next, so you don't think about life or your homework or anything like that. You just think about what's going to happen next.

Crucially, most participants had concealed that they have/had a family member in prison from at least some people in their lives for fear of stigma, judgement and bullying. This meant that some participants had not spoken to a single person outside of their family unit or professionals linked to the justice system about their familial imprisonment to protect themselves and their family members from emotional harm. This meant that children and young people in our study felt incredibly isolated, with the concealment of such information leading to additional stress. They described having to think on the spot, remember what they've said in the past, keep up with past lies, and always know how to answer questions from their friends about their family members. Participants also described the guilt and burden of keeping a secret from friends and lying, in some cases impacting how they feel about themselves:

It's just the lying really and you don't really want to lie your friends. Because that just makes you look like you're a bad person really, if you're just lying and not telling them something.

Kelsey

On the other hand, there were participants for whom it was widely known that they had a family member in prison, due to the impact of media attention and word of mouth, which causes a huge amount of stress, shame and stigma for families of people in prison. For Hannah, the experience of familial imprisonment had lead to a level of acceptance that privacy was not achievable, as well as distrust of people when it comes to sharing information:

They're always listening, because there'll be other ones walking down. Because there'll be one walking up and down here, listening to these. There'll be one walking down on this side, then there'll be one walking down here… We're not really bothered, because it's no different than a phone call. They can just pick the phone up and listen any other time.

Hannah

Because I'm not bothered about people knowing about my dad because look, everybody knows. I can't trust people. If someone knows something then everybody finds out.

Hannah

#### 4.5.2 Supporting Family

Participants discussed concern for family members now taking on greater care‐giving and financial responsibilities as a result of imprisonment (due to, e.g., the loss of a household income, financially supporting the family member in prison, the costs associated with maintaining contact, and the loss of a primary care‐giver) while also managing their own emotions about the loss of a family member through imprisonment. This concern meant that participants were often less likely to discuss their own emotions with family members, as to not further burden them.

It's mostly my mam…It's because whenever she speaks, you know how she gets really emotional? I just worry about if it affects, like how much it does, because if she worries too much then she'll just only think about all that.

Dean

Examples were also shared of participants taking on care‐giving roles for younger siblings, some of whom were not aware that their family member was in prison. The following quote illustrates the emotional weight that Callie carried, along with her mother, in deciding to try to protect her younger siblings from harm through concealment, and the perceived impact that sharing the truth would have on her siblings well‐being:

If we told her [younger sister, 8] she wouldn't believe us and then she would go into a mental breakdown and she wouldn't speak for like three days probably.

Callie

#### 4.5.3 The Impact of Professionals

Those who had friends who were aware of their familial imprisonment shared that they found their support very helpful. However, professional support received from prison staff, social workers, school staff and third sector organisations was not always timely, age‐appropriate, or what the child needed at the time. Our participants generally found school to be an opportune space for support, yet found that teachers were often either unaware or uninformed.

I don't like talking to the school about stuff because you can't trust them.

Hannah

One participant was able to visit and wanted to but a court order meant her mother could not be present in either virtual or physical visits so she was relying on a member of school staff to facilitate a virtual visit, but was met with repeated disappointment which caused her a lot of frustration and stress. Throughout the course of the study, she was waiting for a visit to be organised through the school, and her father was released before it was organised (over 6 months of waiting):

I asked the guidance teacher and she bluntly said to me, “Oh for God's sake, I'll do it in my own time.” She's booked three but cancelled them.

Callie

It is important that professionals working with children who have a family member in prison understand the emotional, mental and physical toll that familial imprisonment can have in order to respond appropriately, to not further stigmatise, and to offer much needed support. There are capacity, staffing and funding issues across sectors for the organisations listed, and we therefore assert the need for greater Government funding for organisations providing vital support to those with a loved one in prison, and that greater policy attention must be paid to the health and wellbeing impacts of familial imprisonment on children and young people.

Some positive examples of youth groups specifically supporting children with a family member in prison were shared by participants. One participant said if they could give one piece of advice to a person in a similar position to them, they would recommend joining a justice‐involved‐focused youth group.

It's got everybody that has a family member in prison, and everyone would go, if you have that, and then you would just get to speak to people that's in your position.

Jodie

However, the majority of young people did not access or have access to such groups due to a combination of limited provision or capacity and, for one participant, concerns about concealment. Thus, children and young people that we spoke to wanted to see big changes in societal attitudes towards prisoners and their families, and a better understanding from their peers and all professionals who work with them, as well as opportunities to spend time with other children in similar circumstances to themselves.

## 5 Discussion

Children and young people with a family member in prison experience a complex mix of uncertainty, instability, grief, and worry. The sudden separation from a family member—often without clear explanation or preparation—can create deep emotional confusion. Meanwhile, uncertainty about the imprisoned family member's wellbeing and the length of their absence can lead to persistent feelings of worry and insecurity. This emotional instability is often intensified by disruptions to everyday life, such as changes in living arrangements, school routines, or financial circumstances, with fear of stigma and associated concealment of circumstances often added to the emotional burden that children and young people experiencing family imprisonment face. This emotional turmoil is often exacerbated for children when their mother is imprisoned [44, 45, 46, 51]. For our participants, these overlapping stressors were harmful and manifested as anxiety for many participants, in both emotional and physical forms—such as trouble sleeping, difficulty concentrating, struggling to sit still, and withdrawal from peers. We posit here that, without appropriate support, this anxiety is more likely to become chronic and—in line with extant literature on childhood adversary and ACE—impact on long‐term emotional and psychological health. Such consequences may be particularly pertinent for children and young people who do not disclose that they are experiencing family imprisonment. In such circumstances, it is not always possible for professionals to tell which children are affected by familial imprisonment. For example, similarly to Flynn & Gor [52], our participants found that teachers, even when well‐meaning, were often either unaware or uninformed. Thus, we assert here that greater awareness amongst ‐ and training of—all professionals who work with children and young people within and beyond the prison context is needed to increase understanding of what children and young people with an imprisoned family member go through, the impact that contact with the system has on them, and the positive impact that kindness and understanding from professionals can have on their experience.

Of course, we have to place this in the context of cuts to public services, including the criminal justice system. Prisons are under immense pressure with decaying buildings, increasing prison populations and serious overcrowding. These issues affect the safety and wellbeing of people in prison and staff, as well as having knock‐on effects on families. In 2025, the House of Commons Justice Committee reported that overcrowding had led to arbitrary prison transfers and reduced access to purposeful activity, education and family contact [53]. These concerns increase the importance of services that support children with a family member in prison and taking a family and relationships focussed approach.

Boundary ambiguity refers to the perception of uncertainty surrounding a loss, and it has been identified as a key predictor of family conflict and symptoms of depression and anxiety [28, 54]. The sense of uncertainty caused by loss and shifting familial and potentially household roles and dynamics is compounded by the fact that the loss associated with imprisonment is often not socially recognised—a concept referred to as disenfranchised grief [27]. As a result, families of people in prison may not receive the social acknowledgement or support typically offered in other forms of loss. As our participants often concealed their familial imprisonment, their support networks were often unavailable to them, as well as limited access to more formal support mechanisms, for fear of stigma and judgement. Importantly, boundary ambiguity does not only concern the absent individual but can extend throughout the broader family and social network, influencing the level of support or isolation experienced. The sense of isolation our participants felt only further increased the need to strengthen family ties and bonding opportunities with their family members in prison, as well as with the wider family.

Thus, from participant narratives, it is clear that, where deemed safe and appropriate, prison visiting is vital for the health and wellbeing of children and families, as it can help to reduce the anxiety and emotional distress associated with familial imprisonment while maintaining family ties. Regular visits provide reassurance that a loved one is safe, support familial attachments, and offer a sense of stability during a time of disruption. Positive contact with their family member can also mitigate against the harmful effects of stress associated with familial imprisonment by offering emotional connection and a sense of routine and normalcy. For this reason, family or children's visit in particular were praised by participants as being how they wished all visits could be, with more freedom of movement and opportunities of activities and bonding. Yet, Woodall & Kinsella [35] highlight that while such visits are welcomed and should be more accessible and available, it is important to recognise that such visit are not a ‘silver bullet’, that prisoners and their families are not homogenous, and a variety of opportunities for support and contact are necessary in order to go so way towards addressing the health and social inequalities faced by people in prison and their families.

As explored in the article, there are current aspects of prison visiting that may cause further harm. Our participants described a system of visiting that lacked consistency, which often involved complex logistical challenges and barriers and, in some cases, exacerbated their stress and anxiety and can be sensorily overwhelming, and at the very least can be boring. Our participants wanted the opportunity to share a ‘proper’ meal together and, as highlighted by Adams et al. [55] and Harman et al. [56], the visiting room offering an otherwise unavailable opportunity for ‘relational bonding’ and ‘family foodcare’ through sharing food, yet this was only possible in one example where a participants' parent was moved to an open prison. Adams et al. [55] and Harman et al. [56] highlight the importance of eating together in family bonding and that the quality of the food shared has an impact on the quality of such a bonding activity, and thus should be treated as such within prison visiting.

Our participants also found the restrictions of movement and physical contact very difficult. In the context of children visiting their mothers in prison, Morgan and Leeson [57] assert the impact of restrictive visiting rules, particularly the lack of movement and physical contact allowed, as having a detrimental impact on the emotions of mothers and their children. Hindt et al. [8] highlight the importance of physical touch and the developmental need for contact without restrictions and barriers in forming and maintaining family bonds and attachment. These restrictions go against policies which emphasise the importance of bonding and maintaining family ties [57]. In discussing symbiotic harms, Condry and Minson [5] describe the political messaging that is communicated to families of people in prison. In our study participants described visuals such as bare walls, guards ‘everywhere’, clothing depicting the status of people in prison, as well as messages communicated through long waiting times, uncomfortable chairs, unappealing and expensive food options, as well as negative attitudes of some staff and a lack of care when designing visit structure and timing. These messages also extended beyond visiting for our participants, seen in trust and hopes being broken by a system which is confusing, inconsistent, and does not centre their needs. For Condry and Minson [5], feelings of unfairness and broken trust as a result of such political messaging impacts families of people in prison in profound ways. The factors that restrict visiting (those that are unavoidable and those that are by design) are harmful to children's wellbeing. There were, however, examples shared of positive examples of good practice in relation to visiting and support offered from within and beyond the prison context which are important to acknowledge. Yet funding and capacity issues often mean support is limited and geographically inconsistent [13].

In response, we propose a re‐design of how families experiencing family imprisonment are supported which places the needs of children and families at its core through the Three Cs Framework. These changes should be informed by a strategic needs assessment and made in partnership with families impacted by imprisonment as well as organisations which support them [13]. We assert that such system overhaul should not consist of the justice system alone but also incorporate health, care and social systems. We also posit that our Three Cs Framework provides a sensible and thoughtful way in which to frame the practical and structural changes children and young people asked for in order to maintain meaningful contact with their family member.

### 5.1 Limitations

Our research does not represent the voices of all children and young people experiencing family imprisonment. Firstly, this study focussed on Scotland, England and Wales, and it is important to recognise that there is limited research focussed on children experiencing family imprisonment in low to middle‐income countries. Within our focus, while we invested a huge amount of time in the recruitment stage of this project as we anticipated that recruitment would be a challenge, we do have limitations in terms of representation within our sample. Our sample is overwhelmingly white British (N = 18), while we know that black and minority ethnic groups are overrepresented in the UK prison population [5]. We attribute this mainly to the populations in the prisons where we had the most access to visits, and this was reflected in the visiting populations. Though it is important to note that the recruitment was undertaken by white researchers which may have acted as a barrier to participation, especially considering the sensitivity of the topic [58]. The vast majority of participants' family members were imprisoned in the male estate (*n* = 17), and although we defined family members broadly (allowing participants to define for themselves), the majority family members who were imprisoned were fathers or step‐fathers/mother's partners. Again, we believe due to greater access to men's prisons for recruitment, as well as a greater male prison populations in the regions of study. Also, while some participants' family member was released during the course of the study, our project did not specifically focus on the period of release. Many of our participants imagined that once release occurred things would ‘go back to normal’ and their health and wellbeing would recover. Further research is needed that ethically and sensitively explores young people's experiences of different aspects of the justice system longitudinally, from arrest to release, including the period of resettlement.

Despite our aim to have a more even split across Northern England and Scotland, the majority were in the North of England (n = 15). Though we had a good retention rate and anticipated some drops outs, it does mean we do not have longitudinal data for all participants. Finally, as the focus of this research was the experiences of visiting with recruitment conducted through prison visitors' centres, this means there is a natural exclusion of children who do not or are not able to visit in our sample. The scope of this project had a visiting focus so this natural exclusion was anticipated in the project design. We do, however, recognise that further work is needed to identify the health and wellbeing impacts of familial imprisonment on those who are not able to visit (e.g., due to legal restrictions) or choose not to visit.

## 6 Conclusion

This qualitative study of the impact of familial imprisonment on children and young people's health and wellbeing illustrated the complex and multifaceted harms that result from familial imprisonment. While not appropriate for all children, our study highlights the importance of strengthening families ties through child‐centred contact for the health and wellbeing of children impacted by imprisonment. In this article we propose a re‐design of how families experiencing family imprisonment are supported, within and beyond the prison walls, which places the needs and rights of children and families at its core. The ‘Three Cs’ framework begins with a focus on redesign of visits and contact with the criminal justice system that is Child‐centred. It then addresses the need for greater Clarity and consistency throughout the process, and finally addresses the need for greater system‐wide compassion, respect and understanding. In Box A, we summarise children and young people's recommendations into a ‘manifesto for change’, which we suggest must sit alongside our framework.

Box A **Children and young people's manifesto for change.**

- Child‐centred contact and visits that are welcoming, relaxed, conducive to bonding and have things to do for all ages. Visits designed to centralise the needs of children will be accessible and beneficial to all, subsequently improving the visiting experience for everyone.
- A more relaxed experience with friendly staff, comfortable chairs, better food and drink, and more colourful decoration; toys, books and (video) games that are of good quality and working properly.
- Timings of visits that are suited to the need of visitors (taking into account public transport timings, school and work).
- An increase in the number of family days/children's visits and other opportunities for bonding with activities (e.g. homework clubs), within and outside of the prison.
- Reduced distances that families have to travel for visits by locating people close to their families.
- Reduced costs of visiting as well as providing consistently available financial support for families to support visiting.
- Free phone calls (for mobile phones and landlines) to promote family bonding and contact.
- Clearer and more welcoming processes and procedures.
- Clearer and consistent rules and attitudes within and between prison sites.
- Support and information throughout the custodial journey for children and young people experiencing familial imprisonment that is both consistent and age‐appropriate to help them to understand the process and to allow space for questions and concerns to be raised.
- Greater care and compassion from all who work within the prison system towards families of people in prison, using a child‐centred approach to visits as well as contact with the system.
- Training to increase awareness and understanding of what children and young people with an imprisoned family member go through, the impact that contact with the system has on them, and the positive impact that kindness and understanding from professionals can have on their experience.
- Greater recognition and understanding for the complex impact that familial imprisonment has on children and young people from all professionals who work with them.
- Greater and consistent support for children experiencing familial imprisonment that is tailored to individual circumstances and needs.
- Greater funding for charities that support the children and families of imprisoned people to increase their vital experienced work.
- Greater provision of targeted youth groups to support children who are touched by the criminal justice system to target isolation, shame and stigma.

## Author Contributions

**Naomi Griffin:** conceptualization (supporting), data collection (lead), formal analysis (equal), methodology (supporting), writing – original draft (lead), writing – review and editing (equal). **Lisa Crowe:** formal analysis (equal), writing – review and editing (equal). **Nancy Loucks:** funding acquisition (supporting), project administration (supporting), supervision (supporting), writing – review and editing (equal). **Shona Minson:** funding acquisition (supporting), project administration (supporting), supervision (supporting), writing – review and editing (equal). **Tracy Shildrick:** funding acquisition (supporting), project administration (supporting), supervision (supporting), writing – review and editing (equal). **Tina Young:** funding acquisition (supporting), project administration (supporting), supervision (supporting), writing – review and editing (equal). **Steph Scott:** conceptualization (lead), formal analysis (equal), funding acquisition (lead), methodology (lead), project administration (lead), resources (lead), writing– review and editing (equal).

## Ethics Statement

Ethical approval for this study was provided by Newcastle University's Faculty of Medical Sciences (FMS) Ethics Committee (Reference: 2346/23630).

## Consent

All participants who took part in this study provided informed written consent.

## Conflicts of Interest

The authors declare no conflicts of interest.

## Data Availability

Research data includes sensitive or confidential information regarding stigma, children and young people and criminal justice system involvement. It is justifiable that the data are not made available.

## Endnotes

1 With the exception of participant 9, who had experience of visiting her father during a previous sentence but was at time of interview facing difficulty visiting due to a court order which prohibited her mother from visiting.

2 Virtual visits are pre‐booked video‐calls with a time limit
